# Role of apolipoprotein E (ApoE) ε4 in cognitive impairment after a stroke: a prospective cohort study

**DOI:** 10.18632/aging.206248

**Published:** 2025-05-08

**Authors:** Jia-Hung Chen, Lung Chan, Chien-Tai Hong, Chaur-Jong Hu, Yi-Chen Hsieh

**Affiliations:** 1 Ph.D. Program in Medical Neuroscience, College of Medical Science and Technology, Taipei Medical University, Taipei, Taiwan; 2 Department of Neurology, School of Medicine, College of Medicine, Taipei Medical University, Taipei, Taiwan; 3 Department of Neurology, Taipei Medical University-Shuang Ho Hospital, New Taipei, Taiwan

**Keywords:** ApoE, cognitive impairment, post-stroke

## Abstract

Although apolipoprotein E (ApoE) ε4 is a well-established risk factor for Alzheimer disease, its role in the development of post-stroke cognitive impairment (PSCI) remains uncertain. In this prospective cohort study, we recruited patients aged ≥20 years who had ischemic stroke within the past 7 days and measured their ApoE genotype. Baseline characteristics, including age, sex, education level, medical history, stroke severity, stroke etiology, and neuroimaging findings were recorded. Cognitive function was evaluated using the Montreal Cognitive Assessment (MoCA) and Clinical Dementia Rating (CDR) at 3 and 12 months post-stroke, with PSCI defined as a MoCA score < 26. After adjusting for confounding factors, the ApoE ε4 allele was not associated with the risk of PSCI at 3 or 12 months post-stroke. Other factors, including age, body mass index, education level, and initial stroke severity, were found to be associated with the risk of PSCI at 3 months. In patients who developed PSCI at 12 months, only education level and the MoCA score at 3 months were significantly associated with the risk of PSCI. Our findings suggest that, aside from traditional risk factors, the ApoE ε4 allele does not contribute to the risk of PSCI at 3 or 12 months post-stroke. Further studies with a larger sample size and longer follow-up are warranted.

## INTRODUCTION

Stroke is the third leading cause of death and the fourth leading cause of disability-adjusted life years worldwide, contributing substantially to post-event disability [1]. Although its impact on physical function is well documented, stroke can also precipitate a diverse range of cognitive challenges. A meta-analysis of 16 hospital-based studies reported that more than 50% of stroke survivors develop neurocognitive disorders following stroke onset [2]. These cognitive deficits encompass disruptions in memory, attention, language, executive functions, and other cognitive processes [3]. Additionally, the severity of post-stroke cognitive impairment (PSCI) considerably varies among individuals and is influenced by a combination of stroke-related characteristics, vascular risk factors, and non-modifiable factors including age, genetic variants, and pre-existing cognitive conditions [4, 5].

The apolipoprotein E (ApoE) genotype is a well-established genetic determinant of cognitive impairment [6]. The ApoE gene is polymorphic and comprises three primary alleles: ε2, ε3, and ε4. Among these, the ApoE ε4 allele is particularly notable due to its strong association with an increased risk of late-onset Alzheimer’s disease (AD), the most common form of dementia [7]. Individuals who carry one copy of the ApoE ε4 allele are at an increased risk of developing AD compared with those who do not. This elevated risk is partly attributed to disruptions in lipid metabolism and transport in the brain, mediated by the ApoE protein [8].

Although PSCI shares some pathological features with AD, the association between the ApoE ε4 allele and PSCI remains underexplored. Studies investigating the risk of PSCI in relation to ApoE ε4 status are limited and have yielded inconclusive results, with some reporting a positive association and others reporting no significant effect [9–11]. To address this uncertainty, we conducted a cohort study of stroke patients and assessed their cognitive function at different time points following stroke onset. This investigation aimed to elucidate whether the presence of the ApoE ε4 allele contributes to an elevated risk of cognitive decline following a stroke.

## RESULTS

### Participant characteristics

**Figure 1:**
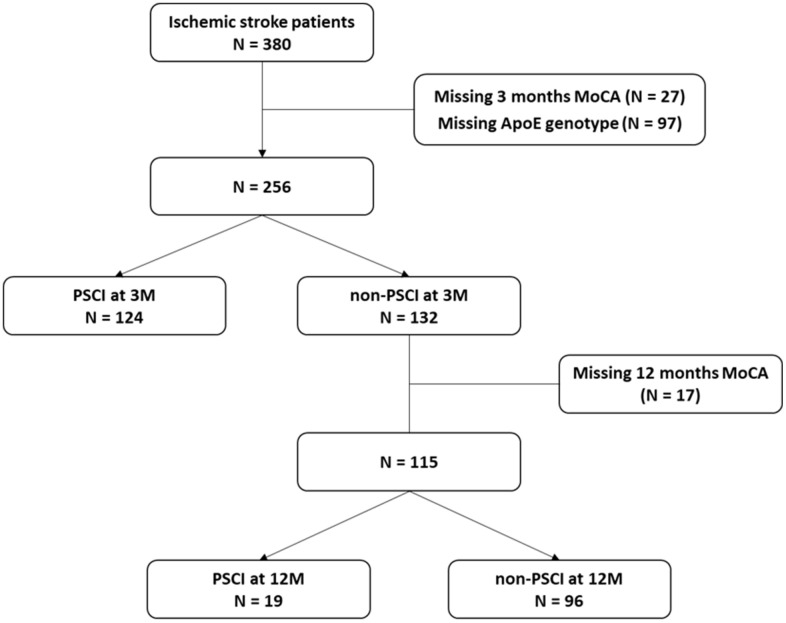
Patient selection flowchart.

As shown in Figure 1, a total of 380 patients were enrolled in the cohort. Of these, 27 were excluded due to missing Montreal Cognitive Assessment (MoCA) scores at 3 months, and an additional 97 were excluded because of missing ApoE genotype results. Thus, 256 patients remained, among whom 124 were classified as having PSCI at 3 months, whereas 132 were not. A comparison of the two groups revealed that patients with PSCI exhibited significantly lower MoCA scores at both 3 months (19.45 ± 6.19 vs. 28.06 ± 2.79, *p* < 0.0001) and 12 months (20.02 ± 7.73 vs. 28.09 ± 1.39, *p* < 0.0001). Additionally, those with PSCI were older (64.06 ± 10.13 vs. 53.39 ± 10.40, *p* < 0.0001), had a lower body mass index (BMI; 25.55 ± 3.56 vs. 26.79 ± 4.57, *p* = 0.0196), and had lower educational attainment (42.98% vs. 8.33%, *p* < 0.0001). They also consumed alcohol less frequently (14.29% vs. 25.81%, *p* = 0.0253) and had higher median National Institutes of Health Stroke Scale (NIHSS) scores (4 vs. 3, *p* = 0.0008). Furthermore, Fazekas scores on brain magnetic resonance imaging (MRI) were higher in patients with PSCI than in those without PSCI (*p* < 0.0001). However, no significant differences were observed between the groups in terms of sex, marital status, ApoE ε4 prevalence, medical history, stroke etiology, or plasma biomarker levels. The participant characteristics are detailed in Table 1.

**Table 1:** Characteristics of participants with and without PSCI at 3 months.

**Variable**	**With PSCI (*n* = 124)**	**Without PSCI (*n* = 132)**	***p***
**Demographics (n/%)**			
Age (mean ± SD)	64.06 (10.13)	53.39 (10.40)	<0.0001
BMI (mean ± SD)	25.55 (3.56)	26.79 (4.57)	0.0196
Male	89 (71.77)	93 (70.45)	0.8159
Education ≤6 years	52 (42.98)	11 (8.33)	<0.0001
Married	74 (81.32)	82 (74.55)	0.2515
Alcohol consumption	17 (14.29)	32 (25.81)	0.0253
Smoking habit	63 (52.94)	72 (57.14)	0.5087
ApoE ε4 carrier	23 (18.55)	22 (16.67)	0.6926
**Medical history (n/%)**			
HTN	97 (78.86)	84 (68.29)	0.0601
DM	50 (40.65)	39 (33.05)	0.2217
Dyslipidemia	85 (70.25)	89 (70.63)	0.9469
Heart disease	29 (24.58)	27 (22.69)	0.7324
**Stroke severity**			
NIHSS score (median/IQR)	4 (3–5)	3 (1–5)	0.0008
**TOAST classification (n/%)**			
Large artery atherosclerosis	30 (26.09)	22 (18.03)	0.4055
Cardioembolism	11 (9.57)	12 (9.84)	
Small vessel occlusion	68 (59.13)	75 (61.48)	
Other determined etiology	2 (1.74)	4 (3.28)	
Undetermined etiology	4 (3.48)	9 (7.38)	
**Fazekas scale**			
Periventricular white matter			
0	23 (22.77)	55 (57.89)	<0.0001
1	32 (31.68)	27 (28.42)	
2	13 (12.87)	4 (4,21)	
3	33 (32.67)	9 (9.47)	
Deep white matter			
0	21 (22.83)	35 (39.33)	0.0009
1	33 (35.87)	41 (46.07)	
2	23 (25.00)	8 (8.99)	
3	15 (16.30)	5 (5.62)	
**Plasma biomarkers (pg/mL)**			
Aß 42	16.20 (1.56)	16.55 (1.60)	0.0915
Aß 40	49.84 (5.19)	47.24 (6.32)	0.0858
Aß 42/40 ratio	0.34 (0.07)	0.39 (0.12)	0.0704
Tau	20.90 (6.61)	22.29 (6.51)	0.0970
BDNF	670.17 (259.98)	716.58 (215.23)	0.3660
Ptau181	3.65 (0.90)	3.81 (1.04)	0.2220
**Cognitive function (mean ± SD)**			
MoCA 3 months	19.45 (6.19)	28.06 (2.79)	<0.0001
MoCA 12 months	20.02 (7.73)	28.09 (1.39)	<0.0001

PSCI, post-stroke cognitive impairment; BMI, body mass index; ApoE, apolipoprotein E; HTN, hypertension; DM, diabetic mellitus; NIHSS, National Institutes of Health Stroke Scale; TOAST, Trial of ORG 10172 in Acute Stroke Treatment; Aß, amyloid beta; BDNF, brain-derived neurotrophic factor; MoCA, Montreal Cognitive Assessment.

### Factors associated with progression to PSCI at 12 months in patients initially classified as non-PSCI at 3 months

Among patients initially classified as non-PSCI at 3 months, 17 were lost to follow-up at 12 months. Of the remaining 115 patients, 19 developed PSCI by 12 months (Figure 1). Patients who subsequently developed PSCI at 12 months had significantly lower MoCA score at 12 months (19.37 ± 8.79 vs. 28.61 ± 1.39, *p* = 0.0002). These individuals demonstrated lower levels of education (26.32% vs. 6.25%, *p* = 0.0107), higher median NIHSS scores (5 vs. 2, *p* = 0.0046), and lower MoCA scores at 3 months compared with those who did not develop PSCI (27.21 ± 1.32 vs. 28.23 ± 1.36, *p* = 0.00033). Conversely, other factors associated with PSCI at 3 months, such as age, BMI, alcohol consumption, and Fazekas scores on brain MRI, did not differ significantly between those who did and did not develop PSCI by 12 months. Additionally, no significant differences were noted in ApoE ε4 prevalence, medical history, stroke etiology, or plasma biomarker levels between the two groups (Table 2).

**Table 2:** Characteristics of participants with and without progression to PSCI at 12 months from non-PSCI at 3 months.

**Variable**	**With progression to PSCI (*n* = 19)**	**Without progression to PSCI (*n* = 96)**	***p***
**Demographics (n/%)**			
Age (mean ± SD)	55.26 (13.47)	53.24 (10.14)	0.4544
BMI (mean ± SD)	27.99 (3.08)	26.59 (4.672.5)	0.2532
Male	14 (73.68)	68 (70.68)	0.8018
Education ≤6 years	5 (26.32)	6 (6.25)	0.0107
Married	10 (61.43)	59 (71.95)	1.0000
Alcohol consumption	2 (10.53)	25 (28.41)	0.1467
Smoking habit	10 (52.63)	48 (53.33)	0.9556
ApoE ε4 carrier	3 (15.79)	15 (15.63)	1.0000
**Medical history (n/%)**			
HTN	15 (78.95)	56 (63.64)	0.2002
DM	7 (36.84)	25 (30.12)	0.5690
Dyslipidemia	15 (78.95)	62 (68.89)	0.3817
Heart disease	4 (21.05)	19 (22.62)	1.0000
**Stroke severity**			
NIHSS score (median/IQR)	5 (3–7)	2.5 (1–4)	0.0046
**TOAST classification (n/%)**			
Large artery atherosclerosis	3 (15.79)	15 (16.85)	0.2119
Cardioembolism	4 (21.05)	7 (7.87)	
Small vessel occlusion	9 (47.37)	58 (65.17)	
Other determined etiology	0 (0)	3 (3.37)	
Undetermined etiology	3 (15.79)	6 (6.74)	
**Fazekas scale**			
Periventricular white matter			
0	6 (46.15)	46 (62.16)	0.3000
1	4 (30.77)	19 (25.68)	
2	0 (0)	3 (4.05)	
3	3 (23.08)	6 (8.11)	
Deep white matter			
0	6 (54.55)	28 (39.44)	0.0970
1	2 (18.18)	33 (46.48)	
2	1 (9.09)	7 (9.86)	
3	2 (18.18)	3 (4.23)	
**Plasma biomarkers (pg/mL)**			
Aß 42	16.42 (1.70)	16.60 (1.64)	0.6848
Aß 40	50.19 (8.10)	46.07 (6.02)	0.2320
Aß 42/40 ratio	0.35 (0.13)	0.40 (0.12)	0.4119
Tau	20.14 (6.95)	22.57 (6.60)	0.1700
BDNF	578.88 (197.68)	747.68 (213.16)	0.0635
Ptau181	3.64 (0.97)	3.83 (1.07)	0.5060
**Cognitive function (mean ± SD)**			
MoCA 3 months	27.21 (1.32)	28.23 (1.36)	0.0033
MoCA 12 months	19.37 (8.79)	28.61 (1.39)	0.0002

PSCI, post-stroke cognitive impairment; BMI, body mass index; ApoE, apolipoprotein E; HTN, hypertension; DM, diabetic mellitus; NIHSS, National Institutes of Health Stroke Scale; TOAST, Trial of ORG 10172 in Acute Stroke Treatment; Aß, amyloid beta; BDNF, brain-derived neurotrophic factor; MoCA, Montreal Cognitive Assessment.

### Multivariate regression analyses of ApoE and other risk factors for PSCI

To investigate the association between ApoE ε4 and PSCI development, we conducted multivariate regression analyses for participants with PSCI at 3 months by using three models (Table 3). Each model was adjusted for various factors to identify significant predictors of PSCI. In Model I and Model II, the presence of ApoE ε4 was not significantly associated with PSCI (odds ratio [OR] = 1.18, *p* = 0.6634 in Model I and OR = 1.22, *p* = 0.6018 in Model II). However, in Model III, which had additional variables adjusted for, the presence of ApoE ε4 was close to significant, but not quite, as a predictor of increased PSCI risk (OR = 3.01, *p* = 0.0609).

**Table 3:** Multivariate analysis results for participants with PSCI at 3 months.

	**Model I**		**Model II**		**Model III**	
	**OR (95%CI)**	***p***	**OR (95%CI)**	***p***	**OR (95%CI)**	***p***
Non-ApoE e4 carrier	1.0		1.0		1.0	
ApoE e4 carrier	1.18 (0.56-2.47)	0.6634	1.22 (0.58-2.60)	0.6018	3.01 (0.95-9.50)	0.0609
Age	1.18 (1.05-2.47)	<0.0001	1.08 (0.05-1.12)	<0.0001	1.09 (1.03-1.15)	0.0017
Education >6 years	0.41 (0.26-0.65)	0.0001	0.41 (0.26-0.67)	0.0004	0.41 (0.21-0.80)	0.0095
Initial stroke severity			1.05 (0.96-1.14)	0.3017	1.25 (1.04-1.50)	0.0183
BMI					0.87 (0.77-0.98)	0.0214
Alcohol consumption					0.84 (0.25-2.87)	0.7795
Fazekas scale					1.37 (0.84-2.25)	0.2075

Model I: Adjusted for age and education level.

Model II: Adjusted for age, education level, and initial stroke severity.

Model III: Adjusted for age, education level, initial stroke severity, BMI, alcohol drinking, and Fazekas scale.

PSCI, post-stroke cognitive impairment; OR, odds ration; ApoE, apolipoprotein E; BMI, body mass index.

Among other risk factors, age was consistently associated with an increased risk of PSCI across all models (OR = 1.18, *p* < 0.0001 in Model I; OR = 1.08, *p* < 0.0001 in Model II; OR = 1.09, *p* = 0.0017 in Model III). Similarly, a higher education level (defined as >6 years of schooling) consistently acted as a protective factor against PSCI across all models (OR = 0.41, *p* = 0.0001 in Model I; OR = 0.41, *p* = 0.0004 in Model II; OR = 0.41, *p* = 0.0095 in Model III). In Model III, initial stroke severity was positively correlated with PSCI (OR = 1.25, *p* = 0.0183), whereas BMI was negatively correlated with PSCI (OR = 0.87, *p* = 0.0214). However, alcohol consumption and the Fazekas scale were not significantly associated with PSCI in Model III (OR = 0.84, *p* = 0.7795 and OR = 1.37, *p* = 0.2075, respectively).

For those who subsequently developed PSCI, multivariate analysis across three models revealed that the presence of ApoE ε4 was not significantly associated with the development of PSCI in any of the models (OR = 0.71, *p* = 0.6464 in Model I; OR = 0.55, *p* = 0.4393 in Model II; OR = 0.83, *p* = 0.8233 in Model III; Table 4). However, a higher education level consistently functioned as a protective factor against the development of PSCI at 12 months, with significant associations observed across all models (OR = 0.33, *p* = 0.0070 in Model I; OR = 0.37, *p* = 0.0182 in Model II; OR = 0.43, *p* = 0.0481 in Model III). Additionally, the MoCA score at 3 months was a significant predictor of subsequent PSCI, with lower scores at 3 months indicating a higher risk of cognitive decline at 12 months (OR = 0.59, *p* = 0.0169).

**Table 4:** Multivariate analysis results for participants who developed PSCI at 12 months.

	**Model I**		**Model II**		**Model III**	
	**OR (95%CI)**	***p***	**OR (95%CI)**	***p***	**OR (95%CI)**	***p***
Non-ApoE e4 carrier	1.0		1.0		1.0	
ApoE e4 carrier	0.71 (0.17–2.96)	0.6464	0.55 (0.12–2.53)	0.4393	0.83 (0.17–4.18)	0.8233
Education >6 years	0.33 (0.14–0.74)	0.0070	0.37 (0.16–0.84)	0.0182	0.43 (0.18–0.99)	0.0481
Initial stroke severity			1.06 (0.96–1.18)	0.2536	1.05 (0.94–1.17)	0.3604
MoCA score at 3M					0.59 (0.38–0.91)	0.0169

Model I: Adjusted for education level.

Model II: Adjusted for education level and initial stroke severity.

Model III: Adjusted for education level, initial stroke severity, and MoCA score at 3M.

PSCI, post-stroke cognitive impairment; OR, odds ration; ApoE, apolipoprotein E; MoCA, Montreal Cognitive Assessment.

Since PSCI could also be evaluated using the Clinical Dementia Rating (CDR), we defined a CDR score ≥ 0.5 as indicative of PSCI and re-tested our model accordingly (Supplementary Tables 1, 2). After multivariate regression analyses, ApoE ε4 was not associated with the risk of PSCI across all models, both at 3 months (OR = 0.80, *p* = 0.5587 in Model I; OR = 0.71, *p* = 0.3756 in Model II; OR = 0.79, *p* = 0.6181 in Model III; Supplementary Table 3) and 12 months (OR = 0.41, *p* = 0.2692 in Model I; OR = 0.35, *p* = 0.2678 in Model II; OR = 0.56, *p* = 0.5682 in Model III; Supplementary Table 4) post stroke.

## DISCUSSION

In this prospective cohort study, we investigated the association between the presence of ApoE ε4 and the development of PSCI. After adjustments for potential confounders, such as age, education level, initial stroke severity, BMI, alcohol consumption, and the Fazekas scale, our findings did not reveal a significant association between ApoE ε4 and PSCI at 3 or 12 months post-stroke. Other factors, including age, BMI, education level, and initial stroke severity, were identified as significant predictors of PSCI development at 3 months. Furthermore, education level and the MoCA score at 3 months were identified as significant predictors of the subsequent development of PSCI at 12 months.

The ApoE ε4 has been associated with various neurodegenerative diseases, including AD, Parkinson’s disease, ischemic stroke, and multiple sclerosis [12–15]. ApoE plays a crucial role in cholesterol transport and lipid distribution within the brain, which may contribute to the pathophysiology of neurodegeneration [16, 17]. The ε4 variant was demonstrated to impair the ability of ApoE to effectively clear Aβ plaques, a hallmark of AD [18]. This dysfunction leads to the accumulation of amyloid plaques in the brain, which are neurotoxic and contribute to cognitive impairment. In patients with small subcortical infarctions, Aβ positivity has been identified as a significant predictor of PSCI development and cognitive decline over 1 year, suggesting that ApoE ε4 may influence PSCI through its role in Aβ accumulation [19]. However, our study did not observe a significant association between ApoE ε4 and the development of PSCI. Additionally, Aβ levels did not differ between individuals with and without PSCI. This inconsistent result may stem from differences in patient groups and methods used to measure Aβ burden, as our study examined blood samples rather than amyloid positron emission tomography scan. Nevertheless. the lack of a difference in blood Aβ levels may explain why our study did not demonstrate an association between ApoE ε4 and PSCI.

Previous research on the association between ApoE ε4 and cognitive decline after stroke is controversial. Some studies have indicated that carriers of the ApoE ε4 are at an increased risk of developing cognitive impairment following a stroke. In the Canadian Study of Health and Aging (CSHA), a higher prevalence of dementia, ranging from 40.6% to 57.6%, was observed in older patients who had both a stroke and the ApoE ε4 allele [20]. A similar association was observed in a small population study in China, which suggested that ApoE gene polymorphism is associated with cognitive impairment in post-stroke patients [21]. However, the CSHA study enrolled patients with an average age of over 80 years, which was older than the average age of around 60 years in our study. Additionally, the study conducted in China included patients with higher NIHSS scores, averaging 8-10, compared to an average score of 4 in our study. Therefore, the results of our study may not be directly comparable to these studies.

Although our study did not support an association between ApoE ε4 and PSCI, the findings were consistent with those of other studies. In a Korean study, a comparison between patients with AD, vascular dementia (VD), and normal controls suggest that ApoE ε4 allele was not more prevalent in the VD patients [22]. A similar finding was observed in a Bahraini cohort, which demonstrated no significant associations between ApoE genotype and cognitive impairment post stroke [23]. Furthermore, data from the Oxford Vascular Study have indicated that the association between ApoE ε4 and PSCI is independent of cerebrovascular burden, potentially mediated by increased neurodegenerative pathology or heightened vulnerability to injury [24]. Therefore, cognitive decline after stroke may not be accelerated by the presence of ApoE ε4, which is consistent with the Chicago Health and Aging Project (CHAP) study, suggesting that cognitive decline before and after stroke was not significantly different among those with the ApoE ε4 allele [25].

In addition to ApoE ε4, several other factors, such as age, BMI, education level, and initial stroke severity, were identified to be significant contributors to the development of PSCI in our study. As expected, advanced age was associated with a higher risk of cognitive impairment post-stroke, consistent with the general age-related decline in cognitive function observed in other stroke populations, particularly among those carrying ApoE ε4 [26]. Lower BMI was also identified as a risk factor for cognitive impairment in the Asian population, which may be mediated by factors such as nutritional deficiencies, reduced brain volume, and poorer metabolic health [27, 28]. Education level, a key determinant of cognitive function, has also been demonstrated to act as a protective factor against cognitive decline, which may be attributed to the cognitive reserve hypothesis [29]. In the Rotterdam Study, individuals with higher education levels exhibited lower risks of developing dementia following a stroke or transient ischemic attack [30]. Consistent with this finding, the present study demonstrated a lower risk of PSCI among patients with more than 6 years of education. Furthermore, in the present study, more severe initial stroke symptoms were associated with a higher risk of developing PSCI, supporting the hypothesis that the extent of brain damage at the time of the stroke directly influences cognitive outcomes [31, 32]. However, further investigation is needed because most patients in the present study had lower NIHSS scores, with an average score of 4.

Interestingly, although alcohol consumption was not revealed as a significant predictor in the multivariate analysis, it was more commonly reported among patients without PSCI. Some studies have suggested that moderate alcohol consumption may exert a protective effect on cognitive function, whereas excessive alcohol consumption could lead to cognitive decline [33, 34]. However, in the present study, the exact quantity of alcohol consumed by each individual could not be measured, and alcohol use was assessed using self-reported data, which may be subject to reporting bias. Patients with cognitive impairment may have underreported their alcohol consumption due to memory difficulties or lack of awareness, whereas those without PSCI may have been more accurate or forthcoming in their reporting. Therefore, further research involving more precise methods for measuring alcohol intake should be considered to elucidate the potential role of alcohol in post-stroke cognitive health.

Although several risk factors were identified as contributors to PSCI at 3 months, the contributions of some of these factors were not significant in those who subsequently developed PSCI at 12 months. A lower education level was consistently associated with the development of subsequent PSCI, whereas age, stroke severity, and ApoE ε4 were not. Notably, the MoCA score at 3 months was specifically associated with subsequent PSCI. These findings suggest that despite ApoE ε4 may accelerate cognitive decline in the early post-stroke phase, its impact may diminish over time. By contrast, factors such as education level and MoCA scores are more significant predictors of long-term PSCI development. This underscores the importance of early and ongoing cognitive monitoring in stroke survivors.

The strength of this study lies in its comprehensive evaluation of multiple risk factors for PSCI, particularly the ApoE ε4 genotype. Additionally, the prospective design enables the observation of changes over time, providing valuable insights into the development of PSCI at both 3 and 12 months post-stroke. However, the study has some limitations that must be addressed. First, the relatively mild stroke severity in our cohort may limit the generalizability of the results to patients with more severe strokes or different stroke subtypes. Second, our study did not assess stroke location or stroke volume in patients, both of which are thought to have a significant impact on the development of PSCI. However, the patients included in our study had relatively low NIHSS scores, suggesting a smaller stroke volume. Additionally, we excluded patients with large or strategic infarcts, which further minimized the potential impact of stroke location in our study. Third, the cutoff level for MoCA used to define PSCI varies across different studies, and MoCA performance may be influenced by education level, making it challenging to establish a precise cutoff. Nevertheless, we used the CDR as an alternative neuropsychological tool to re-analyze the association between ApoE ε4 and PSCI, and this consistently showed no significant association. Finally, unmeasured factors such as other genetic variants, comorbidities, and lifestyle factors may have contributed to residual confounding. Further research is required to explore these variables and enhance the understanding of PSCI in stroke survivors.

In conclusion, our study did not demonstrate an association between ApoE ε4 and the development of PSCI. Other factors, such as advanced age, lower BMI, lower education level, and more severe initial stroke symptoms, were identified as predictors of PSCI development. Additionally, the MoCA score at 3 months was found to be a significant predictor of cognitive decline at 12 months, highlighting the importance of early cognitive assessment and ongoing monitoring in stroke survivors.

## MATERIALS AND METHODS

### Study design and participants

This prospective cohort study investigated cognitive outcomes in patients following acute ischemic stroke. Since 2015, patients aged ≥20 years admitted to Taipei Medical University—Shuang Ho Hospital due to acute ischemic stroke within 7 days of symptom onset were screened for eligibility. Individuals with a history of premorbid cognitive impairment, mood disorders, or neurodegenerative diseases that affected their daily activities were excluded from the study. To focus on post-stroke cognitive trajectories, we also excluded the following patients: (1) those with large infarcts that resulted in immediate consciousness impairment; (2) those with strategic infarcts involving the hippocampus or medial frontal cortex; and (3) those with severe language or physical disabilities precluding neuropsychological assessment. All eligible participants underwent baseline evaluation within 7 days of stroke onset. Follow-up assessments were conducted at the outpatient department at 3 and 12 months post-stroke. This study was approved by the Joint Institutional Review Board of Taipei Medical University (N202104050), and written informed consent was obtained from all participants or their legal guardians.

### Data collection

Baseline characteristics, including age, sex, education level, personal and medical history, stroke severity, and stroke etiology, were collected during enrollment by study-trained nurses and subsequently reviewed by the principal investigators. Stroke severity was assessed using the NIHSS, and stroke etiology was classified according to the Trial of ORG 10172 in Acute Stroke Treatment criteria. Upon admission, all the participants underwent brain MRI to confirm the presence of acute ischemic stroke, as evidenced by diffusion-weighted images. White matter hyperintensities were also evaluated using the Fazekas rating scale. To assess cognitive function, the MoCA and CDR were performed at 3 and 12 months post-stroke. In this study, patients with a MoCA score < 26 at 3 months post-stroke were classified as having PSCI. Additionally, blood samplings were collected upon admission for ApoE genotype analysis and plasma biomarker measurements.

### ApoE genotype and plasma biomarker measurement

Blood samples were collected through venous draw within 7 days of stroke onset. The collected samples were then centrifuged at 1,500 × *g* for 15 min at room temperature, after which plasma from ethylenediaminetetraacetic acid tubes was aliquoted into 0.5-mL microcentrifuge tubes and stored at −80° C until analysis. To determine the ApoE genotype, DNA was extracted using the QIAamp 250 DNA Blood Maxi Kit (QIAGEN, Clayton VIC, Australia) following the manufacturer’s protocol. The ApoE SNPs were genotyped using the TaqMan allelic discrimination assay (Applied Biosystems, Foster City, CA, USA). Plasma levels of amyloid beta (Aβ) 40, Aβ42, and tau were measured using immunomagnetic reduction assays developed by MagQu (New Protein Analysis, Taipei City, Taiwan). Additionally, plasma levels of brain-derived neurotrophic factor were quantified using an enzyme-linked immunosorbent assay with cytokine detection kits (DY248; R&D Systems, Minneapolis, MN, USA).

### Statistical analysis

Data were analyzed using descriptive and inferential statistical methods. Continuous variables are expressed in terms of the mean ± standard deviation, whereas categorical variables are presented as frequencies and percentages. Between-group comparisons were performed using independent *t* tests for continuous variables and chi-square tests for categorical variables. To identify factors associated with the development of PSCI at 3 and 12 months post-stroke, multivariate logistic regression analyses were performed. Three models were constructed for each outcome (PSCI at 3 months and PSCI at 12 months) to account for various potential confounding factors. All statistical analyses were conducted using SAS (version 9.4, Cary, NC, USA). A two-tailed *p*-value of <0.05 was considered statistically significant.

## Supplementary Material

Supplementary Tables
