# Supplementary material for: Role of apolipoprotein E (ApoE) ε4 in cognitive impairment after a stroke: a prospective cohort study
Source: Aging (Albany NY). 2025 May 8;17(5):1261–74. doi: 10.18632/aging.206248 (PMC12151514; doi:10.18632/aging.206248)
Supplement: Supplementary Tables [file aging-17-206248-s001.pdf]

SUPPLEMENTARY TABLES

Supplementary Table 1. Characteristics of participants with and without PSCI at 3 months.

Variable	With PSCI (*n* = 115)	Without PSCI (*n* = 127)	*p*
**Demographics (n/%)**			
Age (mean ± SD)	61.94 (11.91)	55.97 (10.46)	<0.0001
BMI (mean ± SD)	26.44 (4.50)	26.06 (3.74)	0.4951
Male	87(68.50)	85(73.91)	0.3540
Education ≤6 years	44(34.92)	17(15.04)	0.0004
Married	76(77.55)	68(77.27)	0.9639
Alcohol consumption	22(18.49)	29(27.10)	0.1219
Smoking habit	65(54.17)	61(55.45)	0.8446
ApoE ε4 carrier	18(15.93)	20(18.69)	0.5880
**Medical history (n/%)**			
HTN	94(76.42)	73(67.59)	0.1346
DM	52(43.33)	30(28.30)	0.0190
Dyslipidemia	80(65.57)	82(74.55)	0.1371
Heart disease	30(25.86)	21(20.19)	0.3198
**Stroke severity**			
NIHSS score (median/IQR)	4(2-6)	3(1-5)	0.0145
**TOAST classification (n/%)**			
Large artery atherosclerosis	35(29.91)	17(16.19)	0.0130
Cardioembolism	58(49.57)	73(69.52)	
Small vessel occlusion	14(11.97)	7(6.67)	
Other determined etiology	5(4.27)	1(0.95)	
Undetermined etiology	5(4.27)	7(6.67)	
**Fazekas scale**			
Periventricular white matter			
0	29 (34.12)	40 (47.06)	0.0194
1	20 (23.53)	28 (32.94)	
2	11 (12.94)	5 (5.88)	
3	25 (29.41)	12 (14.12)	
Deep white matter			
0	19 (24.68)	31 (38.75)	0.0304
1	26 (33.77)	32 (40.00)	
2	16 (20.78)	11 (13.75)	
3	16 (20.78)	6 (7.50)	
**Plasma biomarkers (pg/mL)**			
Aß 42	16.42 (1.28)	16.59 (1.47)	0.3559
Aß 40	48.92 (4.89)	49.39 (6.39)	0.7704
Aß 42/40 ratio	0.34 (0.06)	0.36 (0.11)	0.3254
Tau	21.09 (4.61)	22.04 (5.91)	0.1783
BDNF	695.29 (250.20)	690.06 (255.13)	0.9348
Ptau181	3.65 (0.84)	3.78 (1.01)	0.2998
**Cognitive function (mean ± SD)**			
MoCA 3 months	21.87 (6.90)	26.65 (3.13)	<0.0001
MoCA 12 months	21.43 (7.96)	26.72 (4.73)	<0.0001

PSCI, post-stroke cognitive impairment; BMI, body mass index; ApoE, apolipoprotein E; HTN, hypertension; DM, diabetic mellitus; NIHSS, National Institutes of Health Stroke Scale; TOAST, Trial of ORG 10172 in Acute Stroke Treatment; Aß, amyloid beta; BDNF, brain-derived neurotrophic factor; MoCA, Montreal Cognitive Assessment.

Supplementary Table 2. Characteristics of participants with and without progression to PSCI at 12 months from non-PSCI at 3 months.

Variable	With progression to PSCI (*n* = 26)	Without progression to PSCI (*n* = 70)	*p*
**Demographics (n/%)**			
Age (mean ± SD)	57.54 (10.54)	54.41 (10.40)	0.1956
BMI (mean ± SD)	25.89 (4.05)	26.26 (3.64)	0.6911
Male	18(69.23)	55(78.57)	0.3407
Education ≤6 years	3(11.54)	9(13.24)	1.0000
Married	13(65.00)	40(76.92)	0.3039
Alcohol consumption	5(19.23)	18(28.57)	0.3600
Smoking habit	12(46.15)	39(60.00)	0.2293
ApoE ε4 carrier	2(8.33)	12(17.65)	0.3423
**Medical history (n/%)**			
HTN	17(70.83)	41(63.08)	0.4955
DM	8(36.36)	15(23.08)	0.2219
Dyslipidemia	20(83.33)	50(74.63)	0.3850
Heart disease	2(9.09)	16(25.00)	0.1391
**Stroke severity**			
NIHSS score (median/IQR)	3.5(2-4.5)	3(1-5)	0.4633
**TOAST classification (n/%)**			
Large artery atherosclerosis	4(20.00)	7(10.45)	0.4906
Cardioembolism	15(75.00)	49(73.13)	
Small vessel occlusion	0(0.00)	5(7.46)	
Other determined etiology	0(0.00)	0(0.00)	
Undetermined etiology	1(5.00)	6(8.96)	
**Fazekas scale**			
Periventricular white matter			
0	11 (47.83)	24 (47.06)	0.2508
1	5 (21.74)	20 (39.22)	
2	2 (8.70)	2 (3.92)	
3	5 (21.74)	5 (9.80)	
Deep white matter			
0	7 (30.43)	19 (41.30)	0.0435
1	8 (34.78)	20 (43.48)	
2	4 (17.39)	7 (15.22	
3	4 (17.39)	0 (0.00)	
**Plasma biomarkers (pg/mL)**			
Aß 42	16.58 (2.11)	16.66 (1.35)	0.8769
Aß 40	51.08 (7.64)	46.78 (5.13)	0.1381
Aß 42/40 ratio	0.34 (0.13)	0.39 (0.10)	0.3467
Tau	21.19 (6.20)	22.45 (5.97)	0.3795
BDNF	675.11 (274.78)	684.29 (247.17)	0.9247
Ptau181	3.33 (0.96)	3.92 (1.06)	0.0182
**Cognitive function (mean ± SD)**			
MoCA 3 months	25.81 (4.01)	27.09 (2.69)	0.1418
MoCA 12 months	24.60 (6.08)	27.86 (2.22)	0.0145

PSCI, post-stroke cognitive impairment; BMI, body mass index; ApoE, apolipoprotein E; HTN, hypertension; DM, diabetic mellitus; NIHSS, National Institutes of Health Stroke Scale; TOAST, Trial of ORG 10172 in Acute Stroke Treatment; Aß, amyloid beta; BDNF, brain-derived neurotrophic factor; MoCA, Montreal Cognitive Assessment.

Supplementary Table 3. Multivariate analysis results for participants with PSCI at 3 months.

	Model I		Model II		Model III	
	**OR (95%CI)**	***p***	**OR (95%CI)**	***p***	**OR (95%CI)**	***p***
Non-ApoE e4 carrier	1.0		1.0		1.0	
ApoE e4 carrier	0.80 (0.39–1.68)	0.5587	0.71 (0.33–1.52)	0.3756	0.79 (0.31–1.99)	0.6181
Age	1.03 (1.00-1.06)	0.0245	1.04 (1.01–1.07)	0.0111	1.04 (1.01–1.09)	0.0278
Education >6 years	0.46 (0.22–0.95)	0.0368	0.60 (0.28–1.29)	0.1885	0.52 (0.21–1.31)	0.1669
Initial stroke severity			1.10 (1.00–1.20)	0.0421	1.09 (0.98-1.22)	0.1205
Diabetes					1.95 (0.90–4.22)	0.0883
Fazekas scale					1.16 (0.81–1.66)	0.4130

Model I: Adjusted for age and education level.
Model II: Adjusted for age, education level, and initial stroke severity.
Model III: Adjusted for age, education level, initial stroke severity, diabetes, and Fazekas scale.
PSCI, post-stroke cognitive impairment; OR, odds ration; ApoE, apolipoprotein E.

Supplementary Table 4. Multivariate analysis results for participants who developed PSCI at 12 months.

	Model I		Model II		Model III	
	**OR (95%CI)**	***p***	**OR (95%CI)**	***p***	**OR (95%CI)**	***p***
Non-ApoE e4 carrier	1.0		1.0		1.0	
ApoE e4 carrier	0.41 (0.08–2.00)	0.2692	0.35 (0.06-2.23)	0.2678	0.56 (0.08–4.17)	0.5682
Education >6 years	1.02 (0.25–4.21)	0.9805	1.01 (0.22–4.62)	0.9907	1.11 (0.13–9.65)	0.9256
Initial stroke severity			1.12 (0.95–1.32)	0.1762	1.20 (0.98–1.48)	0.0743
Fazekas scale					2.58 (1.19–5.60)	0.0165

Model I: Adjusted for education level.
Model II: Adjusted for education level and initial stroke severity.
Model III: Adjusted for education level, initial stroke severity, and Fazekas scale at 3M.
PSCI, post-stroke cognitive impairment; OR, odds ration; ApoE, apolipoprotein E.
